# Supplementary material for: Adenoviral-Vectored Centralized Consensus Hemagglutinin Vaccine Provides Broad Protection against H2 Influenza a Virus
Source: Vaccines (Basel). 2022 Jun 10;10(6):926. doi: 10.3390/vaccines10060926 (PMC9229510; doi:10.3390/vaccines10060926)
Supplement: Supplementary file 1 [file vaccines-10-00926-s001.zip › vaccines-1741409-supplementary.pdf]

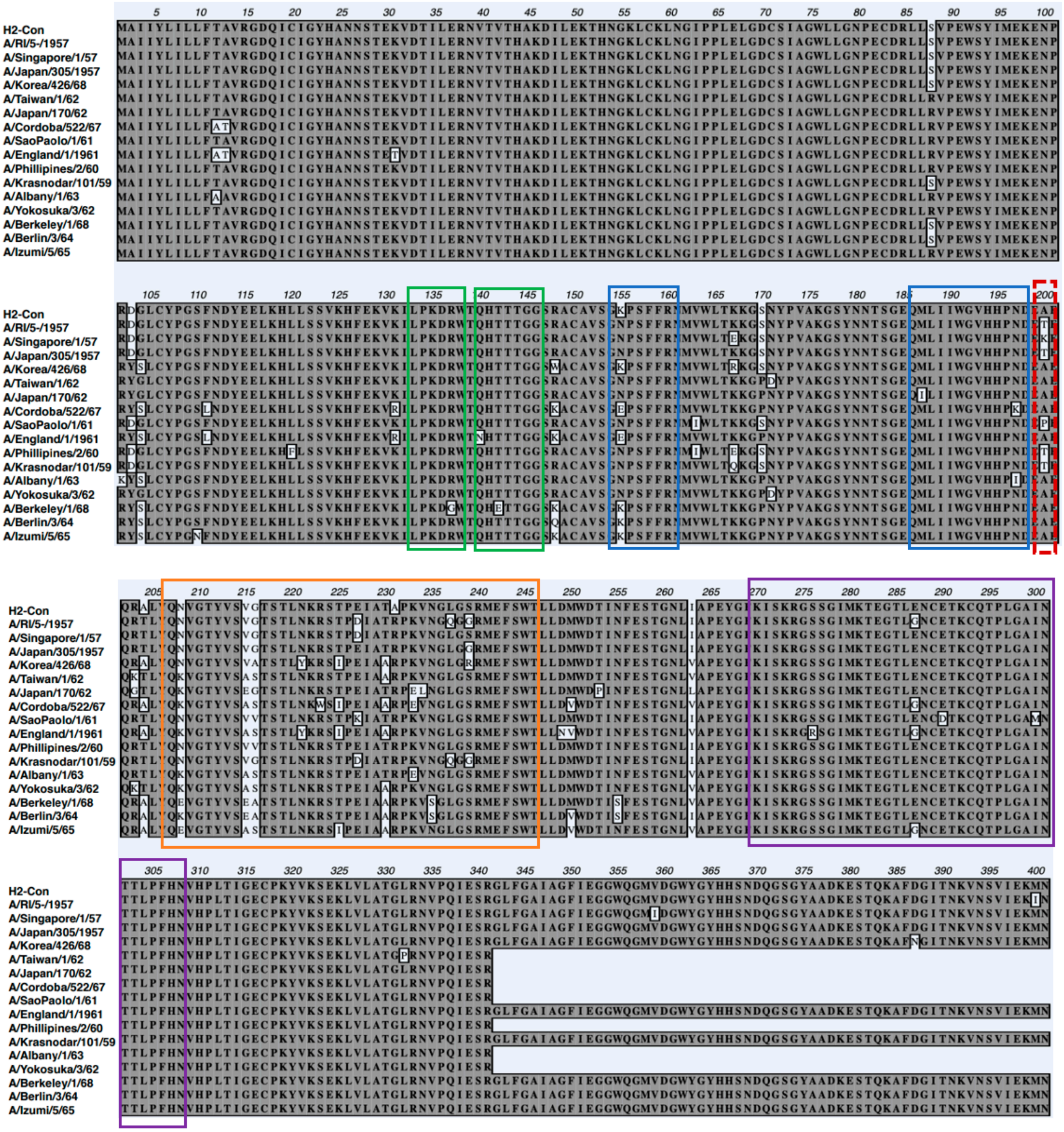
5 10 15 20 25 30 35 40 45 50 55 60 65 70 75 80 85 90 95 100
H2-Con
A/RI/5-/1957
A/Singapore/1/57
A/Japan/305/1957
A/Korea/426/68
A/Taiwan/1/62
A/Japan/170/62
A/Cordoba/522/67
A/SaoPaolo/1/61
A/England/1/1961
A/Phillipines/2/60
A/Krasnodar/101/59
A/Albany/1/63
A/Yokosuka/3/62
A/Berkeley/1/68
A/Berlin/3/64
A/Izumi/5/65
105 110 115 120 125 130 135 140 145 150 155 160 165 170 175 180 190 195 200
205 210 215 220 225 230 235 240 245 250 255 260 265 270 275 280 285 290 295 300
305 310 315 320 325 330 335 340 345 350 355 360 365 370 375 380 385 390 395 400

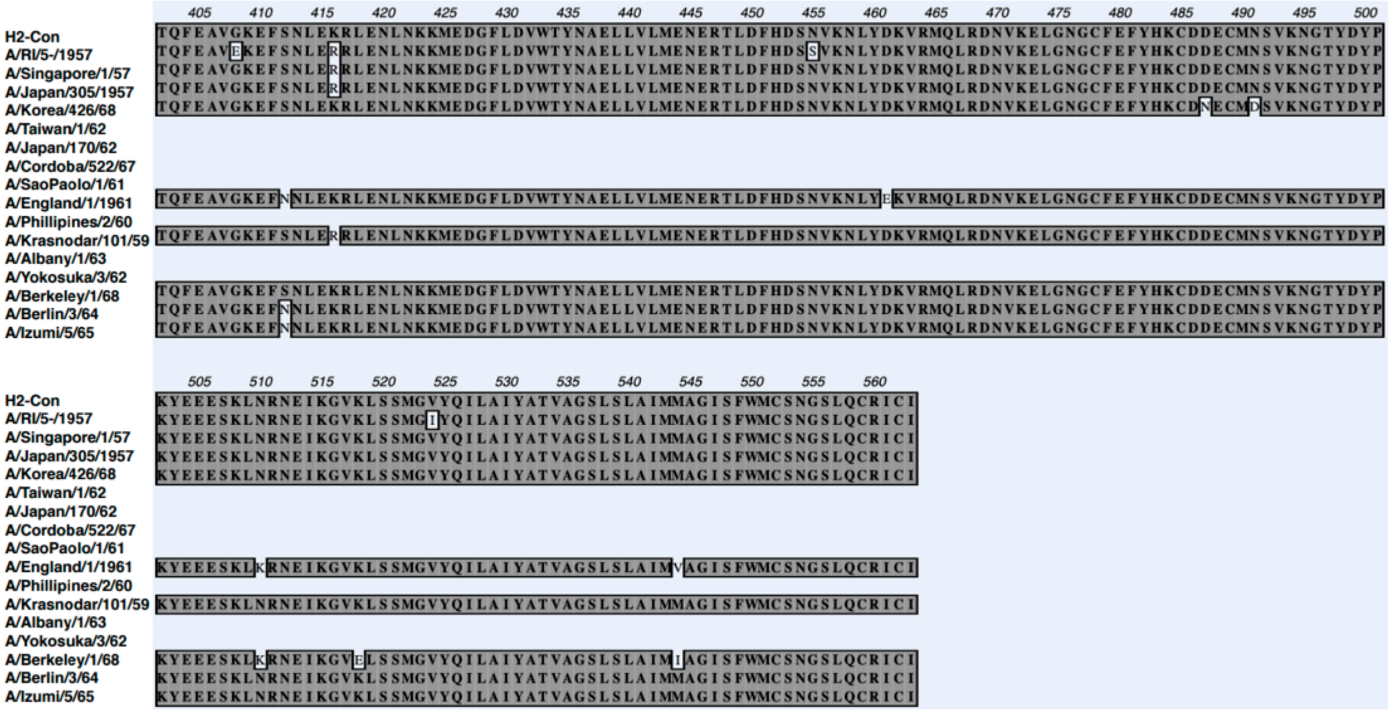

Figure S1. Sequence alignment of the H2 consensus (H2-Con) immunogen, representative strains used in the immunogen design, and study strains used in immune correlates and challenge. Sequences were downloaded from GenBank and aligned with ClustalW and visualized using the protein alignment tool in MacVector. Antigenic sites are shown in colored boxes and are as follows: A (green), B (blue), C (purple), and D (orange) [1,2]. A dotted, red box at amino acid position 200 indicates the alanine-to-threonine (A200T) mutation seen in Japan/57 and RI/57 that is located distally proximal to antigenic sites B and D. The black arrow denotes an arginine residue that may serve as a scaffold for a glycosylation site on these antigenic regions.

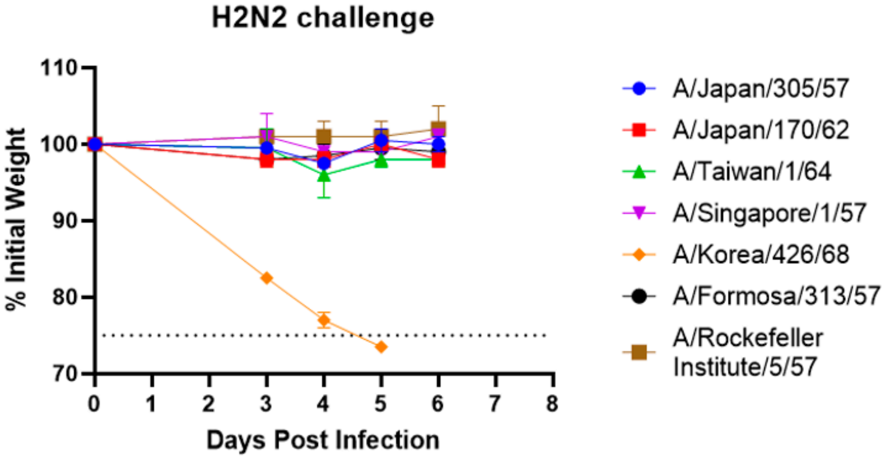

Figure S2. Initial measure of lethality of representative strains in mouse models. Six- to eight-week-old female BALB/c mice (n = 2) were intranasally infected with seven different egg-grown human H2 strains under ketamine and xylazine-induced anesthesia. Mice were infected with 20 µL of allantoic fluid and observed for 6 days post-infection (dpi) to determine clinical signs of disease. Mice that lost ≥ 75% of their initial body weight were humanely euthanized by CO_2 asphyxiation and cervical dislocation.

1. Reneer, Z.B.; Ross, T.M. H2 influenza viruses: Designing vaccines against future H2 pandemics. *Biochem. Soc. Trans.* **2019**, *47*, 251–264. https://doi.org/10.1042/bst20180602.

2. Tsuchiya, E.; Sugawara, K.; Hongo, S.; Matsuzaki, Y.; Muraki, Y.; Li, Z.-N.; Nakamura, K. Antigenic structure of the haemagglutinin of human influenza A/H2N2 virus. *J. Gen. Virol.* **2001**, *82 Pt 10*, 2475–2484. https://doi.org/10.1099/0022-1317-82-10-2475.
